# Treatment of Inverted Toe-Nail

**Published:** 1874-10

**Authors:** W. Hukill


Treatment of Inverted Toe-Nails—*By Dr. W. Hukill.*—Every practitioner, probably, is acquainted with this affection. Every one knows, also, that the various plans of treatment generally pursued are very unsatisfactory. Removal of the nail, or a part of it, is now generally considered the only certain remedy. This procedure is, to say the least, barbarous. The purpose of this article is, however, not to discuss the accepted modes of treatment, but to recommend a mode of treatment that has been employed by the writer for about four years, which has been highly satisfactory. It is, simply to apply the muriated tincture of iron to the nail and the surrounding ulcerated and granulated surface, once or twice a day, with a camel's hair pencil. As a general rule, to apply it once a day, at bed-time, will be sufficient. The ulcerated surface heals with astonishing rapidity, and the nail assumes its normal appearance, making a complete cure, in most cases, in a few weeks. Since I commenced using this remedy, I have done nothing else in such cases. Paring and cutting the corners of the nail usually do more harm than good. I need not attempt to speak of the *modus operandi* of the remedy, the object being merely to recommend a trial of it to others.—*Medical and Surgical Reporter—August.*

